# Plasmin releases the anti-tumor peptide from the NC1 domain of collagen XIX

**DOI:** 10.18632/oncotarget.2849

**Published:** 2015-01-21

**Authors:** Jean-Baptiste Oudart, Sylvie Brassart-Pasco, Alexia Vautrin, Christèle Sellier, Carine Machado, Aurelie Dupont-Deshorgue, Bertrand Brassart, S. Baud, Manuel Dauchez, Jean-Claude Monboisse, Dominique Harakat, François-Xavier Maquart, Laurent Ramont

**Affiliations:** ^1^ Université de Reims Champagne-Ardenne, CNRS UMR 7369 (Matrice Extracellulaire et Dynamique Cellulaire, MEDyC), Reims, France; ^2^ Université de Reims Champagne-Ardenne, Institut de Chimie Moléculaire de Reims, CNRS UMR N°7312, Faculté de Pharmacie, Reims, France; ^3^ CHU de Reims, Laboratoire Central de Biochimie, Reims, France; ^4^ Plateau de Modélisation Moléculaire Multi-échelle, UFR Sciences Exactes et Naturelles, Université de Reims Champagne-Ardenne, Reims, France

**Keywords:** Collagen XIX, NC1 domain, Plasmin, Proteolysis, Tumor invasion, type I β-turn

## Abstract

During tumor invasion, tumor cells degrade the extracellular matrix. Basement membrane degradation is responsible for the production of peptides with anti-tumor properties. Type XIX collagen is associated with basement membranes in vascular, neuronal, mesenchymal and epithelial tissues. Previously, we demonstrated that the non-collagenous NC1, C-terminal, domain of collagen XIX [NC1(XIX)] inhibits the migration capacities of tumor cells and exerts a strong inhibition of tumor growth. Here, we demonstrate that plasmin, one of the most important enzyme involved in tumor invasion, was able to release a fragment of NC1(XIX), which retained the anti-tumor activity. Molecular modeling studies showed that NC1(XIX) and the anti-tumor fragment released by plasmin (F4) adopted locally the same type I β-turn conformation. This suggests that the anti-tumor effect is conformation-dependent. This study demonstrates that collagen XIX is a novel proteolytic substrate for plasmin. Such release may constitute a defense of the organism against tumor invasion.

## INTRODUCTION

The basement membrane is a complex structure mainly composed of laminins, nidogens, heparan sulfate proteoglycans (perlecan and/or agrin), and type IV collagen. More recently, associations between basement membrane and minor collagens such as type XVIII, XV or type XIX were reported and named basement membrane zone [1, 2]. These collagenous components associated with the basement membrane represent a dynamic interface between this structure and the stroma. Basement membrane has long been described as a simple architectural cell support. However, it exerts many important biological functions and plays key role in many physiological and pathological situations [3, 4]. Proteolysis of basement membrane components generates peptides, which regulate cell activity. The term of «matrikine» is commonly used to design such extracellular matrix macromolecule-derived peptides [5]. For example, the NC1 domain of the α3(IV) collagen chain (tumstatin) exerts anti-tumorigenic and anti-angiogenic activities [6]. It was previously shown that MMP-9 might release tumstatin from type IV collagen *in vivo* [7].

The collagens constitute a very large and complex family of glycoproteins. This family consists in 28 members. They are classified according to their supramolecular structure and biological function. They may be classified as fibril-forming collagens, fibril-associated collagens with interrupted triple helix (FACIT), network-forming collagens, multiplexins, transmembrane collagens and others [8].

Several matrikines derived from collagens IV or XVIII are now proposed as diagnostic and prognostic markers in diseases [9, 10] or for therapeutic use [6, 11, 12, 13]. Recently, we developed an ELISA against the NC1 domain of type XIX collagen and showed that NC1(XIX) was present in the human sera [14, 15].

Type XIX collagen is one of the minor basement membrane-associated collagens. It was discovered in 1992, isolated from rhabdomyosarcoma cell lines and named collagen Y [16]. This discovery was completed in 1993 by Myers et al who gave it the name of RH collagen [17]. It is a 400 kDa homotrimer, composed by the association of three α1(XIX) chains. Each chain comprises 1142 residues, including six non-collagenous domains (NC1-NC6) separated by five collagenous domains (Col1-Col5) [17, 18, 19]. This collagen presents high structural homology with other types of collagens IX, XII, XIV, XVI. These collagens are part of the FACIT collagen family. Therefore, the collagen XIX was classified as a FACIT collagen [18, 20]. The human gene sequence was characterized in 1997 [21, 22]. Type XIX collagen expression is ubiquitous during embryogenesis. In adults, it is mainly localized in the basement membrane zone of vascular, neuronal, mesenchymal and epithelial tissues, associated with type IV and/or type XVIII collagens [19, 23]. Loss of collagen XIX precedes basement membrane invasion in ductal carcinoma of the female breast [24]. The non-collagenous (NC1) C-terminal domain of type XIX collagen, composed of 19 amino acids, inhibited the migration and invasion capacities of tumor cells *in vitro* [25]. It also exerted a strong inhibition of *in vivo* tumor growth in an experimental murine melanoma model. Moreover, it inhibited tumor angiogenesis *in vitro* and *in vivo* [26]. Accordingly, NC1(XIX) collagen domain could be considered as a new anti-tumor and anti-angiogenic matrikine.

Mechanisms leading to the cleavage of type XIX collagen and to the release of NC1(XIX) have never been studied. Interestingly, a potential sequence for plasmin cleavage, due to the presence of an arginine residue, is present at the beginning of the NC1(XIX) domain. Plasmin is implicated in the cleavage of many extracellular matrix components, or in the activation of matrix metalloproteinase (MMP) zymogens. It is an important serine proteinase, with a wide range of physiological functions and pathological implications [27]. It hydrolyses Arg-Xaa or Lys-Xaa peptide bonds. The proteolytic cleavage of the inactive plasminogen by tissue-type plasminogen activator (tPA) or urokinase-type plasminogen activator (uPA) generates plasmin [28].

The aim of this study was to investigate if plasmin could release NC1(XIX) from the α1(XIX) collagen chain. For that purpose, we submitted a peptide derived from the α1(XIX) chain to plasmin proteolysis *in vitro*. We showed that an active anti-tumor fragment of the NC1(XIX) peptide was rapidly released from the α1(XIX) chain. We identified the cleavage sites and sequenced the different proteolytic fragments. By using different amino acid substitutions in the plasmin cleavage site, we demonstrated that the Arg^1122^ residue was crucial for plasmin cleavage.

## RESULTS

### Cleavage of the C-terminal end of collagen XIX by plasmin and characterization of released peptides

Plasmin specifically cleaves extracellular matrix and basement membrane components. To evaluate whether plasmin could cleave the C-terminal domain of type XIX collagen, we used a synthetic peptide (P36) composed of the last 36 residues of the human α1(XIX) chain, homologous to that previously described by Boudko et al [29]. The P36 peptide contained the last 17 amino-acids of the α1(XIX) helical Col1 domain (SPGAPGPQGPPGPSGRC) and the totality of the C-terminal NC1(XIX) domain (NPEDCLYPVSHAHQRTGGN).

**Figure 1:**
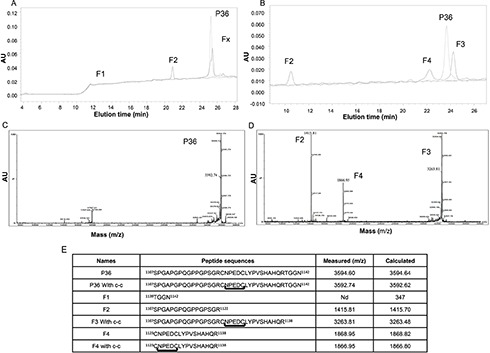
HPLC separation of plasmin degradation products **(A)** P36 peptide was submitted to plasmin digestion at 37°C (E:S = 1:50). The digestion products were separated by HPLC on a Waters analyzer (C_18_ reverse-phase column) by method 1. Three main peaks were separated (F1, F2 and Fx). The non-digested peptide P36 peptide is shown in dotted line. **(B)** By using method 2, the Fx peak was further fractionated and provided two peaks, F3 and F4. The non-digested peptide P36 is shown in dotted line. **(C and D)** MALDI-ToF MS characterization of the peptides released by plasmin digestion. **(C)** The intact P36 peptide analysis revealed a 3592.74 Da major peak, which matched the expected P36 peptide molecular weight. **(D)** After incubation of P36 peptide with plasmin, the plasmin digestion products consisted of 3 major peaks (F2, F3 and F4) of 1415.81 Da, 3263.81 Da and 1866.95 Da respectively. The F2 1415.81 Da peak matched the expected mass of the SPGAPGPQGPPGPSGR Col1 domain of P36 peptide, while the F4 1866.95 Da peak matched the expected CNPEDCLYPVSHAHQR major part of the NC1(XIX) domain of P36 peptide. The F3 3263.81 peak matched the expected entire P36 peptide sequence without the four C-terminal residues (TGGN). **(E)** Plasmin cleavage products of the collagen XIX derived-P36 peptide.

P36 peptide was submitted to plasmin digestion for 16 h. The molar ratio of plasmin to P36 peptide was 1:50. The digestion products were separated by HPLC using the method 1. The absorbance peak (A_214 nm_) of P36 peptide was detected at 25 min with method 1 (Figure 1A) or at 23.5 min with method 2 (Figure 1B). HPLC analysis of the digestion products showed that plasmin hydrolysis generated 3 main peaks: F1, F2, and Fx (Figure 1A). F1 peak was eluted at 11.5 min, F2 at 21 min and Fx at 25.5 min, respectively. The Fx peak was further fractionated using the method 2. It provided two peaks, F3 and F4, with a retention time 24.5 and 22 min, respectively (Figure 1B).

The plasmin digestion products were characterized by MALDI-ToF MS analysis. The analysis of intact P36 peptide revealed a 3592.74 Da major peak, which matched the expected P36 Peptide molecular weight (Figure 1C).

The major plasmin digestion products (F2, F3, and F4) of the P36 peptide corresponded to 1415.81 Da, 3263.81 Da and 1866.95 Da, respectively (Figure 1D). The 1415.81 Da F2 peak matched the expected mass of the SPGAPGPQGPPGPSGR Col1 domain of P36 peptide. The 1866.95 Da F4 peak matched the expected 16 first amino-acid residue sequence (CNPEDCLYPVSHAHQR) of the NC1(XIX) domain. The 3263.81 Da F3 peak matched the expected entire P36 peptide sequence without the four last C-terminal residues (TGGN) (Figure 1D). The F1 peak seemed to correspond to the C-terminal TGGN residues.

MALDI-ToF MS sequence analysis confirmed the *in vitro* plasmin cleavage of P36 peptide at 2 sites. The first plasmin cleavage occurred at Arg1138-Thr1139 and released the 4 C-terminal amino-acid residues of P36 peptide (peak F1, TGGN). The second cleavage occurred at Arg1122-Cys1123 and released the major part of NC1(XIX) domain (peak F4, CNPEDCLYPVSHAHQR). Amino acid sequences of all the plasmin cleavage products are summarized in Figure 1E. MALDI-ToF MS analyses also demonstrated that the P36 peptide, F3 and the F4 fragment formed an intra-chain disulfide-bond between Cys^1123^ and Cys^1128^ in solution over the time (Figure S1).

### Kinetic analysis of P36 peptide cleavage and inhibition by aprotinin

**Figure 2:**
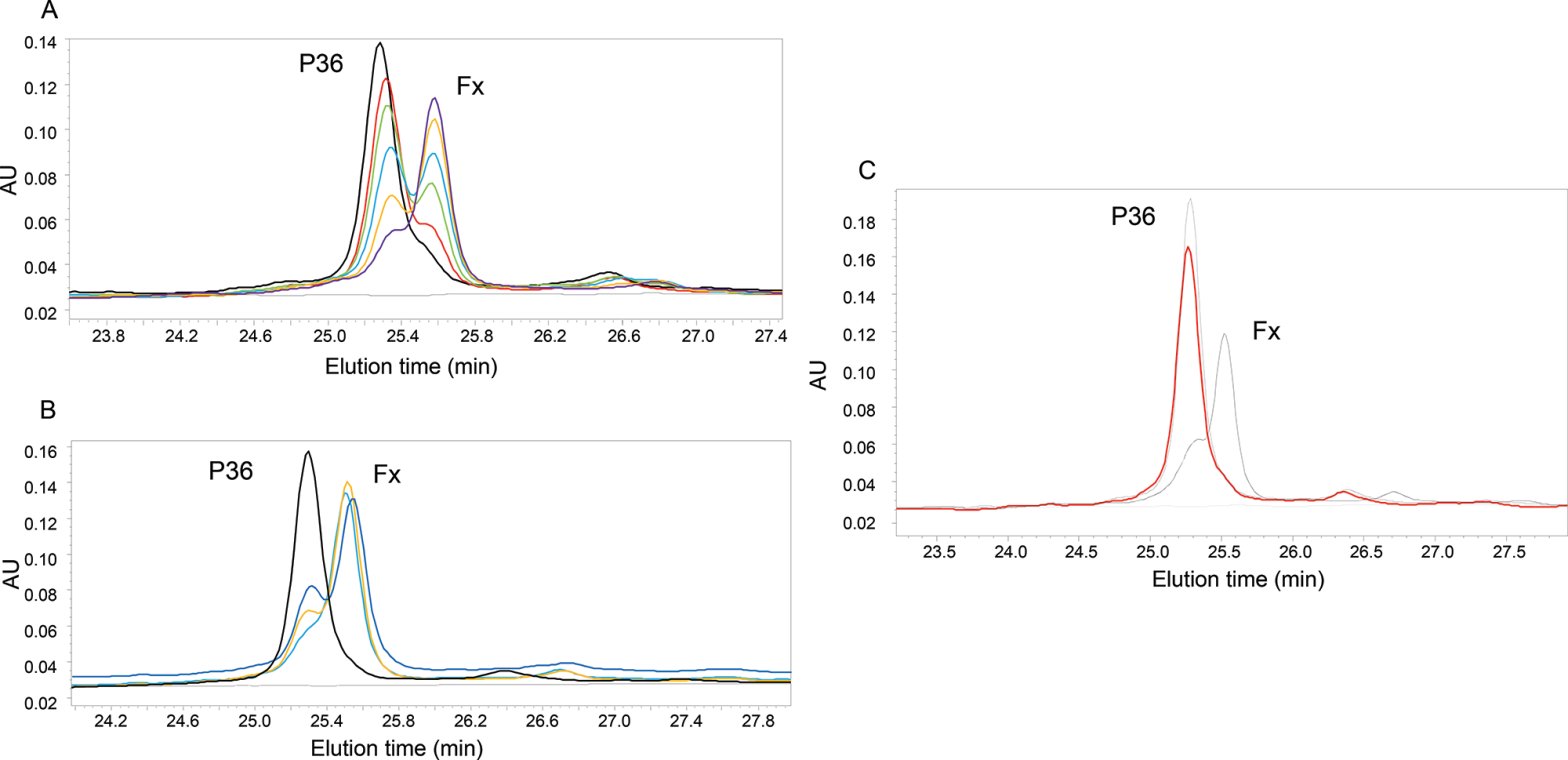
Analysis of P36 peptide cleavage by plasmin **(A)** Dose-dependent digestion of P36 peptide by plasmin: P36 peptide was incubated overnight at 37°C with different plasmin concentrations: 1:50(purple); 1:100(orange); 1:150(blue); 1:200(green): 1:250(red); 1:400 (black). **(B)** Kinetic analysis of the P36 peptide cleavage by plasmin : Kinetics data were obtained by incubating P36 peptide with plasmin (ES = 1:50 at 37°C) 0, 5 min, 30 min, 60 min. HPLC was done with method 1. Only P36 and Fx peaks are shown on the graph. P36 peptide was cleaved as soon as the first 5 minutes of incubation, whereas Fx appeared and increased gradually with time: 0 (black), 5 min (purple), 30 min (orange) and 60 min(blue). Cleavage of P36 by plasmin is inhibited by aprotinin. **(C)** Aprotinin (10^−6^M) was added to the medium during the incubation of P36 peptide with plasmin 16 h at 37°C. We observed that the digestion was completely blocked, without any release of Fx, confirming the implication of plasmin. P36: dotted line; P36 + plasmin: Black line; P36 + plasmin + aprotinin 10^−6^ M: Red line.

The digestion of P36 peptide by plasmin was dose-dependent (Figure 2A). Kinetic analysis of the P36 peptide cleavage by plasmin was performed by incubating P36 with plasmin during 0, 5 min, 30 min or 60 min. HPLC analysis was done with method 1. The digestion of P36 peptide by plasmin was time-dependent (Figure 2B). P36 peptide was cleaved as soon as the first 5 min of incubation whereas plasmin digestion products (Fx) appeared gradually (Figure 2B). K_M_ and Kcat were 73.04 nM, and 0.077s^−1^ respectively and the ratio Kcat/K_M_ was 1506.5 M.s^−1^.

To confirm plasmin involvement in the P36 peptide cleavage, aprotinin was added to the medium during the incubation of P36 with plasmin. 10^−5^ M (pointed grey) and 10^−6^ M (red) aprotinin concentrations completely abolished P36 peptide plasmin digestion whereas 10^−7^ M aprotinin concentration was not sufficient to prevent it (black) (Figure 2C).

### Arg^1122^ P36 peptide substitution suppresses the proteolysis of NC1(XIX) domain by plasmin

**Figure 3:**
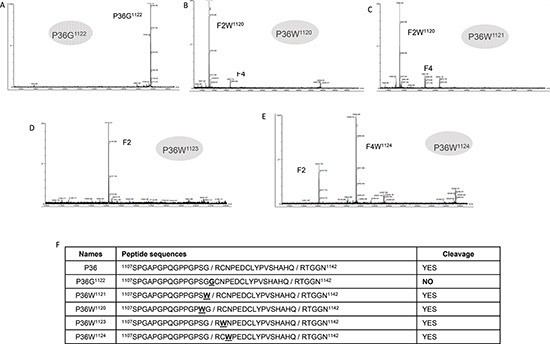
MALDI-ToF MS characterization of the substituted peptide after incubation with plasmin **(A)** MALDI-ToF MS characterization of the substituted R^1122^ to G^1122^ P36 peptide after incubation with plasmin (E:S 1:50, 37°C overnight). No cleavage was observed. **(B)** MALDI-ToF MS characterization of the substituted S^1120^ to W^1120^ P36 peptide after incubation with plasmin (E:S 1:50, 37°C overnight). Complete digestion was observed. **(C)** MALDI-ToF MS characterization of the substituted G^1121^ to W^1121^ P36 peptide after incubation with plasmin (E:S 1:50, 37°C overnight). Complete digestion was observed. **(D)** MALDI-ToF MS characterization of the substituted C^1123^ to W^1123^ P36 peptide after incubation with plasmin (E:S 1:50, 37°C overnight). Complete digestion was observed. **(E)** MALDI-ToF MS characterization of the substituted N^1124^ to W^1124^ P36 peptide after incubation with plasmin (E:S 1:50, 37°C overnight). Complete digestion was observed. **(F)** Panel summarizing the cleavage of modified peptides by plasmin.

Five substituted peptides were synthesized to study the plasmin cleavage site at Arg^1122^ and to determine the amino acids crucial for plasmin cleavage (Figure 3). The Arg^1122^–> Gly substituted P36G^1122^ peptide was no longer cleaved by plasmin. This confirms that Arg^1122^ was crucial for plasmin cleavage (Figure 3A). On the contrary, substitution of the two preceding (P36W^1121^, P36W^1120^) or the two following residues (P36W^1123^, P36W^1124^) still allowed P36 peptide cleavage by plasmin (Figures 3B–3E). These results show that only Arg^1122^ is necessary for the cleavage of P36 peptide by plasmin, whereas the two preceding and the two following residues are not.

### Melanoma cell tPA activates plasmin cleavage of P36, which can be inhibited by tPA siRNA

**Figure 4:**
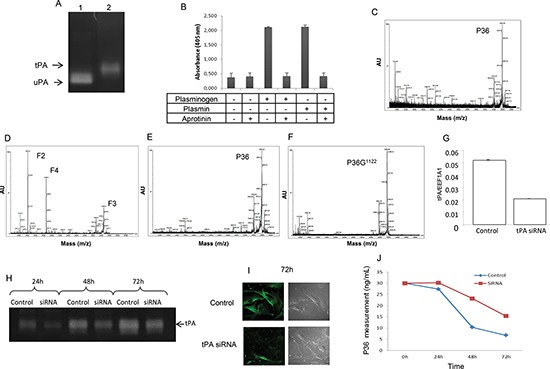
Analysis of P36 peptide cleavage by plasmin in cell culture Plasminogen/plasmin activation system in SKMEL28 melanoma cell cultures. **(A)** Zymography analysis showing that SKMEL28 cells secrete tPA in the extracellular medium (lane 2). No expression of uPA was found. Lane 1 corresponds to uPA positive control. **(B)** Plasmin activity was measured in the SKMEL28 culture medium using a plasmin Chromogenic substrate S-2251 from Chromogenix, with or without addition of plasminogen and/or aprotinin. **(C and D)** MALDI-ToF MS analysis of the SKMEL28 culture medium. **(C)** In the absence of added plasminogen, no cleavage of P36 peptide occurred. **(D)** In presence of added plasminogen in the cell culture medium, P36 peptide disappeared and F2, F3 and F4 peptides were released by plasmin proteolytic cleavage. **(E)** When aprotinin was added to the culture medium supplemented with plasminogen, no cleavage of P36 occurred. **(F)** Substitution of Arg^1122^ in P36 peptide (P36G^1122^) suppressed its cleavage by plasmin. SiRNA (tPA) transfection inhibited the cleavage of P36 peptide. **(G)** Cell transfection with siRNA specific to human tPA decreased tPA mRNA expression. **(H)** Zymography analysis showing that tPA activity was decreased after siRNA (tPA) transfection. **(I)** Immunocytofluorescence experiments showing that tPA protein expression at the melanoma cell surface was decreased after siRNA (tPA) transfection. Left panels: immunocytofluorescence data. Right panels: phase contrast microscopy. **(J)** P36 peptide measurement in the culture medium of SKMEL28 cells transfected with siRNA (tPA). The proteolysis of P36 peptide by plasmin in cell cultures was decreased after siRNA (tPA) transfection.

SKMEL28 melanoma cells were cultured with or without plasminogen and/or aprotinin. These cells secreted tPA into the extracellular medium (Figure 4A). In the presence of plasminogen, cell-generated-plasmin hydrolyzed the chromogenic substrate (Figure 4B). Aprotinin completely inhibited plasmin activity (Figure 4B). In the absence of plasminogen, no release of F2, F3, and F4 was observed (Figure 4C). When plasminogen was added to the culture medium, cell-generated plasmin released the F2, F3 and F4 fragments (Figure 4D). Aprotinin abolished this release (Figure 4E), as well as the Arg^1122^ substitution (Figure 4F).

Cell transfection with siRNA specific to human tPA decreased (−60%) tPA mRNA expression (Figure 4G). Zymography (Figure 4H) and immunocytofluorescence (Figure 4I) studies demonstrated that tPA protein expression was also decreased. Under our experimental conditions, siRNA transfection also decreased the proteolysis of P36 peptide by plasmin in cell culture medium (−56%) (Figure 4J).

### Plasmin-released F4 peptide inhibits melanoma cell migration and melanoma tumor growth

**Figure 5:**
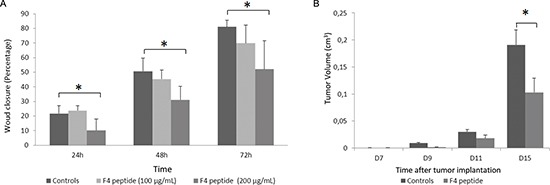
F4 peptide inhibits melanoma cell migration and tumor growth **(A)** Measurement of the wound closure in scratch wound assay, demonstrating that wound closure was significantly delayed when cells were incubated with F4 peptide after 24 h (−53%, *p* < 0.05), 48 h (−39%, *p* < 0.05) or 72 h (−36%, *p* < 0.05) of incubation. Histogram represents the mean ± sd of five experiments; *, *p* < 0.05, significantly different from controls (Mann and Withney non parametric *U*-test). **(B)** F4 peptide inhibits tumor growth in murine melanoma model *in vivo*. Tumor size was measured at days 7, 9, 11 and 15. Tumor volumes were determined according to *v* = 1/2 A x B^2^, where A denotes the largest dimension of the tumor and B represents the smallest dimension. Tumor volume was significantly lower at day 15^th^ (−30%) in F4 peptide-injected mice vs controls. Histogram represents the mean ± sem of ten mice; *, *p* < 0.05, significantly different from controls (Mann and Withney non parametric *U*-test).

In a scratch wound test F4 peptide (200 μg/mL) decreased wound closure after 24 h (−53%, *p* < 0.05), 48 h (−39%, *p* < 0.05) or 72 h (−36%, *p* < 0.05) of incubation (Figure 5A).

Similarly, in an *in vivo* murine melanoma model, F4 peptide significantly inhibited tumor growth at day 15^th^ (−30%, *p* < 0.05) (Figure 5B).

### Molecular modeling studies of P19 and F4 peptides both display a type I β-turn at the N-terminus

**Figure 6:**
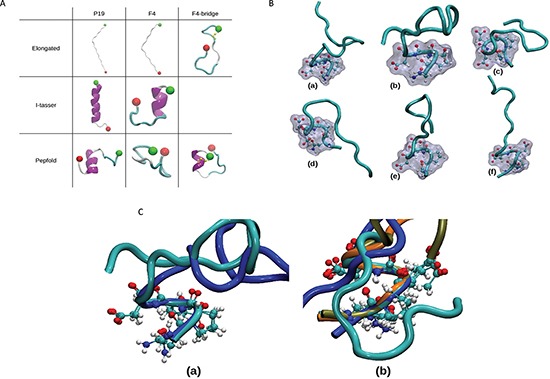
Molecular dynamics simulations: setup and highlight of NPED turn **(A)** Starting conformations of the MD simulations. The different starting conformations of the 8 independent MD simulations are represented using the New-cartoon drawing method. Colors indicate the local secondary structure (white = coil, cyan = turn, magenta = helix), N and C-termini are represented by green and red ball respectively, and the atoms constituting the disulfide bridge are displayed in yellow. **(B)** NPED type I β-turn. From the different simulations, snapshots were extracted when the NPED residues form a type I β-turn : for P19 peptides (a and b) as well as for F4 peptides without (c and d) or with a disulfide bridge (e and f). The backbone is represented using the New-cartoon drawing method and the atoms of the NPED residues are represented using the CPK drawing method and the surface method. **(C)** Superimposition of NPED type 1 β-turn in P19 and F4 peptides. Structural alignment of the P19 peptides (a) and F4 peptides (b) displaying NPED type I β-turn. The alignments were conducted using the positions of the alpha carbons of the NPED residues. The different colours of the backbone correspond to structures extracted from different simulations. (a) P19 generated with I-tasser in blue and P19 generated with Pepfold in cyan. (b) F4 with I-tasser in cyan, F4 generated with Pepfold in blue, F4 with disulfide bridge in tan and F4 with disulfide bridge generated with Pepfold in orange.

Visualization of the different trajectories showed that both P19 and F4 peptides, independently of the starting conformation (displayed in Figure 6A), adopted a small amount of hydrogen secondary structure (β-bridge, β-sheet or helix), low number of β-bridge, β-sheet or helix local structure. Globally, the peptides adopted mostly random structures. These observations were confirmed by the analysis of the local secondary structures performed using the DSSP (Define Secondary Structure of Proteins) algorithm as implemented in the GROMACS package [30]. In all simulations, along the 200 ns, the coil local structure proportion was above 40%. The fractions of bends and turns were more important when starting from conformations generated with the Itasser or the Pepfold servers. The presence of the disulfide bridge between the two cystein residues in the F4 peptides also promoted the formation of turns.

We also determined the localization and the geometry of the β-turns (Table S1). For the 8 MD simulations, the sequences of type I β-turns occurring more than 5% along the whole simulation (1000 occurrences given the 20001 total snapshots for each simulation) are displayed. A first observation was that the peptides whose starting conformation was not an elongated one showed more sequences of type I turns. Moreover, the type I NPED turn was found in 6 MD simulations out of 8 (75% of the simulations). In the case of F4 peptide with disulfide bridge, this local structure was explored more than 60% of the time. We extracted one snapshot from each of these 6 MDs. We could then highlight the geometry of the NPED type I turn (Figure 6B). In addition, for each type of peptide (P19 or F4), we superimposed the type-I turns (Figure 6C).

## DISCUSSION

During tumor invasion, tumor cells invade surrounding normal tissue. In cancer progression, two major classes of proteases play a crucial role, the matrix metalloproteinase (MMP) family and the plasminogen activation system [31]. For migration and invasion, tumor cells degrade the extracellular matrix, and cross vascular epithelial basement membranes. Therefore, matrix metalloproteinases (MMP) and the plasminogen/plasmin system cleave basement membrane components into multiple fragments released into the tumor microenvironment [32].

Collagen XIX is a minor collagen associated with basement membranes. It can be proteolysed during tumor invasion. We previously demonstrated that its 19 amino acids NC1(XIX) domain exerts anti-tumor and anti-angiogenic properties on melanoma. In the present study, we demonstrated that plasmin may release an anti-tumor fragment of NC1(XIX). Plasmin is largely involved in tumor invasion, particularly in melanoma [33, 34].

Previous studies on other basement membrane collagens have clearly demonstrated that collagen NC1 domains are released during tumor invasion. For instance, endostatin, the NC1 domain of type XVIII collagen, is released by many proteases such as MMP-3, 7, 9, 12, 13, 20 [14, 35, 36], or cathepsins B, L, S, V [37, 38, 39]. Endostatin exerts significant anti-tumor properties in number of experimental cancer models [7, 43] and reduces metastatic potential of cells [38]. Tumstatin, the NC1 domain of type IV collagen is generated *in vivo* by MMP-9 [7]. It exerts anti-tumor and anti-angiogenic effects [6]. Fragments of basement membrane collagens, such as endostatin or tumstatin, are present in biological fluids (blood, urine, cerebrospinal fluid...) [11, 14, 15, 41].

In preliminary experiments, we studied the potential role of MMPs in releasing the anti-tumor NC1(XIX) domain. Neither MMP-1, MMP-2, MMP-9 nor MT1-MMP were capable to cleave the P36 peptide and to release an active fragment of the NC1(XIX) domain (data not shown). Beside the MMP family, plasminogen/plasmin system is largely involved in melanoma progression and metastasis [42, 43]. Therefore, we decided to study if plasmin could release the anti-tumor NC1 XIX domain.

Our *in vitro* experiments demonstrated that plasmin cleaved the C-terminal part of collagen XIX and generated a peptide (F4) constituted of the 16 first amino acids of its NC1 domain. The peptide possessed the entire minimal active sequence of the anti-tumor NC1(XIX) domain. The enzymatic activity of plasmin on collagen XIX, as demonstrated by its K_M_ and Kcat, was comparable to those reported in previous studies relative to collagen degradation by proteolytic enzymes [44, 45]. The cleavage was time- and concentration-dependent. Aprotinin, a specific inhibitor of serine proteases, particularly plasmin, inhibited the cleavage. Cleavage sites were localized after the two arginine residues, Arg^1122^ and Arg^1138^. The substitution of arginine Arg^1122^ completely inhibited the release of the F4 peptide by plasmin. This confirms the specificity of the cleavage. No other adjacent amino acid was necessary for enzyme-substrate recognition, since their substitution did not inhibit the cleavage. Furthermore, siRNA (tPA) decreased the proteolysis of NC1(XIX) domain by plasmin in melanoma cell cultures. The decreased tPA expression by tumor cells inhibits the cleavage of NC1(XIX) by plasmin.

In solution, the P36 and F4 peptides spontaneously and rapidly (less than 1 h) formed an intra-chain disulfide bond between cystein C^1123^ and C^1128^. Previous studies on the entire molecule of collagen XIX have also shown that the C-terminal domain was capable of forming intrachain disulfide bond, while other collagens of the FACIT family make interchain bonds [18]. The disulfide bond allowed the formation of a natural loop. The latest might stabilize the anti-tumor sequence and might potentiate the anti-tumor activity. Similar loop formation in a tumstatin-derived cyclopeptide designed in our laboratory demonstrated that such a constraint loop stabilized its active conformation [46, 47].

The F4 peptide was generated not only *in vitro*, but also *ex vivo* in melanoma cell cultures. The addition of the P36 peptide to the culture medium of SKMEL28 melanoma cells induced its cleavage by the plasminogen/plasmin system from the cells. These cells secreted tPA, which cleaved plasminogen into plasmin, which, in turn released the F4 peptide containing the active anti-tumor sequence.

Finally, using computer simulations, we investigated the local structures adopted by F4 and P19 peptides. Especially, when the starting conformations of MD simulations where the ones issued from online servers (I-tasser or Pepfold), we observed the presence of a type I NPED β-turn. In the case of F4 peptides with a disulfide bridge, the amount of this type of turn was significantly increased. Combined with the experimental results, these observations suggest that the NPED active conformation, found at the N-terminus of F4 and P19 peptides, is crucial for anti-tumor activity. Indeed, as it can be observed on Figure 6B and Figure 6C, when the type I NPED is formed, the side chains of the corner residues (PE) are exposed and then totally accessible for interactions.

**Figure 7:**
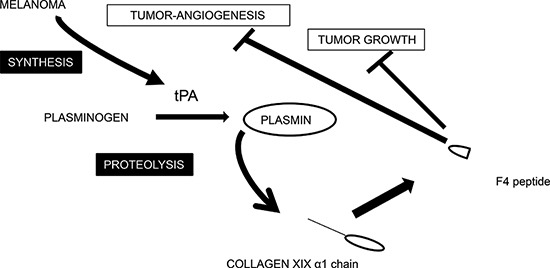
Tentative scheme for integrating the interplay between collagen XIX and melanoma cells Schematic representation of collagen XIX cleavage by the plasminogen/plasmin system in melanoma cell culture. The plasminogen activator tPA is secreted by melanoma cells and tPA activates plasminogen into plasmin. Plasmin cleaves type XIX collagen and releases the anti-tumor F4 fragment from the NC1(XIX) domain.

Collectively, our results strengthen the hypothesis that plasmin cleavage of the NC1(XIX) domain may occur *in vivo* during tumor invasion. It may constitute a mechanism of defense against cancer dissemination and metastasis. A tentative scheme for integrating the interplay between collagen XIX and melanoma cells is shown in Figure 7.

## MATERIALS AND METHODS

### Reagents

Qiagen RNeasy™ kit was from Qiagen (Courtaboeuf, France). Maxima First Strand cDNA synthesis kit and DreamTaq Green PCR Master Mix were from Thermo-Fisher Scientific (Villebon sur Yvette, France). Monoclonal anti-tPA antibody (2A153 clone) was from Abcam (Cambridge, UK). Alexa-488-conjugated secondary antibody was from Invitrogen (Thermo Fisher Scientific, Villebon sur Yvette, France). Immu-mount was from Thermo Scientific Shandon (Thermo Fisher Scientific, Villebon sur Yvette, France). Plasminogen was from Calbiochem, distributed by Millipore (Molsheim, France). Peptides were purchased from Proteogenix (Schiltigheim, France). Their respective sequences are presented in Table S2.

### Cell culture

Human melanoma cell line (SKMEL28) was purchased from the American Type Culture Collection (ATCC, Manassas, VA). Cells were grown in Dulbecco's Modified Eagle's medium (DMEM) supplemented with 10% Fetal Bovine Serum (FBS), 1% penicillin and streptomycin (PenStrep^®^, Gibco^®^, France) in Nunclon^®^ 75 cm^2^ flasks (Dutscher, Brumath, France) at 37°C in a humid atmosphere (5% CO_2_, 95% air). Murine melanoma B16F1 cells, were a generous gift of Dr. M. Grégoire (INSERM UMRS 419, Nantes, France). They were grown in RPMI 1640 medium supplemented with 5% FBS in Nunclon 25 cm^2^ flasks at 37°C in a humid atmosphere (5% CO_2_, 95% air). All culture media and reagents were purchased from Life Technologies (Invitrogen, Strasbourg, France).

### Animals and *in vivo* experiments

C57Bl6 mice (average body weight: 16–18 g) were purchased from Harlan France (Gannat, France). The *in vivo* experiments were conducted according to the ethical guidelines of the Centre National de la Recherche Scientifique (CNRS). *In vivo* melanoma model was previously described (Ramont et al., 2007). Briefly, 2.5 × 10^5^ B16F1 cells, pre-incubated (30 min) with either control medium or F4 peptide, were subcutaneously injected into the left side of 10 syngenic C57BL6 mice. Treatments were re-injected intraperitoneally at day 7 and 14. Tumor size was measured regularly.

### Scratch wound assay

SKMEL28 cells (5 × 10^4^ per well) were seeded in 24-well plates and cultivated to confluence in DMEM supplemented with 5% FBS. The cell layer was then wounded with a sterile 100 to 1000 μL pipet tip and re-incubated in fresh culture medium in the absence or presence of F4 peptide. After 24 h, 48 h or 72 h, cells were photographed and the size of the remaining wound measured.

### Plasmin activity measurement

After washing with DPBS, cells were incubated in DMEM with/without 20 μL plasminogen (40 μg/mL) and/or 20 μL plasmin (10 μg/mL) and/or 20 μL plasmin chromogenic substrate (250 μg/mL). Hydrolysis of the S-2251 chromogenic substrate (Chromogenix, distributed by Hyphen Biomed, Neuville sur Oise, France) was measured at 405 nm at different time [41].

### Gelatin/plasminogen zymography

SKMEL28-conditioned media were analyzed on SDS-polyacrylamide gels containing 1 mg/mL gelatin and 10 μg/mL plasminogen. After electrophoresis, gels were incubated overnight at room temperature in 100 mM glycine buffer, 5 mM EDTA, pH 8.0. Gelatinolytic activity resulting from plasminogen activation was evidenced by white lysis zones after gel staining with G250 Coomassie Brilliant Blue.

### P36 peptide digestion *in vitro*

Lyophilized plasmin with a specific activity of 10 U/mg (Merck, Darmstadt, Allemagne) was dissolved in sterile water to a concentration of 1 mg/mL. In control sample, P36 peptide (6.79 × 10^−4^ M) was incubated overnight at 37°C in a Tris-HCl buffer (150 mM NaCl, 5 mM CaCl_2_, 50 mM Tris-HCl, pH 7.5). To generate proteolytic fragments, P36 peptide (1.25 × 10^−9^ M) was incubated at 1:50 molar ratio with plasmin (2.56 × 10^−11^ M) 16 h at 37°C in the same Tris HCl buffer, with or without the presence of 10^−6^ M aprotinin. Aliquots were analyzed by HPLC as described below.

### Kinetic analysis of enzymatic activity

Dose-dependent digestion of P36 peptide by plasmin was measured by incubating P36 peptide 16 h at 37°C with different plasmin concentrations. Time-dependent analysis of P36 peptide cleavage by plasmin was obtained by incubating P36 with plasmin (E:S 1:50 at 37°C) during different time. Cleavage of the peptide was monitored by HPLC using method 1.

Michaelis constant (K_M_) and first-order rate constant of substrate peptide turnover (Kcat) were obtained by digestion of P36 peptide (8, 16, 32, 64 nM), for 30 min at 37°C in the presence of 1.25 nM plasmin. Cleavage of the peptide was monitored by HPLC. From these measurements, Kcat and K_M_ were calculated using double-reciprocal plot of 1/[S] versus 1/v.

### P36 peptide digestion in cell cultures

SKMEL28 were incubated in DMEM with 20 μL plasminogen (40 μg/mL) and/or 20 μL plasmin (10 μg/mL) and/or aprotinin (10^−5^ M) and P36 peptide. Samples were analyzed using HPLC and Matrix Assisted Laser Desorption Ionisation – Time of flight protocol (MALDI-ToF) as described below.

### High pressure liquid chromatography (HPLC)

HPLC samples (20 μL of each sample with 30 μL of 0.1% trifluoroacetic acid) were injected on a Waters Alliance 2695 HPLC system, equipped with a photodiode array 2996 PDA detector, fraction collector III (WFC III) and the Empower^TM^ chromatography software for data acquisition (Milford, MA, USA). The column used was a EC 250/4 Nucleosil reversed phase (5 μm particle size, 100 Å pore size, 4 × 250 mm) which was protected by a guard column CC 8/4 Nucleosil 300-5 C4 (Macherey-Nagel, Düren, Germany). The injection volume was 20 μL and detection was performed at λ = 214 nm. Eluants were 0.1% trifluoroacetic acid in water (A) and 0.1% trifluoroacetic acid in 80% acetonitrile (B). The analyses followed a linear gradient program with a flow at 0.8 mL/min. Two method conditions were used:

- Method 1: 0–5 min 100% A; 5–35 min 45% A −55% B; 35–41 min 0% A − 100% B; 41–50 min 100% A.

- Method 2: 0–40 min 85% A − 15% B; 40–41 60% A − 40% B; 41–46 min 0% A − 100% B; 46–55 min 85% A − 15% B.

### Matrix assisted laser desorption ionisation – Time of flight (MALDI – ToF)

Solutions of matrix α–cyano-4-hydroxycinnamic acid (CHCA) was prepared as a saturated solution in ACN/water (1/1; v/v) containing 0.1% TFA (10 mg/mL). Samples were prepared at a concentration of 10 pmol/μL in water containing 0.1% TFA. Typically, 1 μL of the matrix was pipetted on the MALDI target plate. Then 1 μL of analyte was added and air-dried for MALDI-ToF MS analysis.

MS experiments were performed using micromass MALDI_TM_–LR time-of-flight mass spectrometer (Waters MS technologies, Manchester, UK) equipped with a nitrogen UV laser (337 nm wavelength), reflectron optics, a fast dual micro-channel plate (MCP) detector and a high magnification camera system. Positive ion spectra were acquired in reflectron mode. The following voltages were applied: pulse 1940 V, MCP 2380 V, suppression 500, flight tube 12000 V, reflectron 5200 V. A time lag focusing (TLF) delay of 680 ns was used between the time of laser pulse and the application of the accelerating voltage. Samples were analyzed in reflectron acquisition mode in the mass range 550-4000 Da. Mass calibration was performed using a peptide mixture composed of Bradykinin fragment 1–5 (573.31 Da), human Angiotensin II (1046.54 Da), Neurotensin (1672.92 Da), ACTH clip (2465.20 Da), bovine Insulin ß-chain oxidized (3494.65 Da) and bovine Insulin (5730.61 Da).

### siRNA transfection

siRNA specific to human tPA (FlexiTube GeneSolution for PLAT) was purchased from Qiagen (Courtaboeuf, France). The siRNA target different regions of the tPA mRNA: 1st siRNA target sequence (5′-cagcatatttatagcaatcca-3′), 2nd siRNA target sequence (5′-aaggagcaagccgtgaattta-3′), 3rd siRNA target sequence (5′-caggaaagacggattgcatta-3′) and 4th siRNA target sequence (5′-cagcagcgcgttggcccagaa-3′). Exponentially growing SKMEL28 cells were transfected with siRNA pools (20 nM) using lipofectamine™ 2000. Decrease of tPA mRNA expression was assessed by RT-qPCR at 48 h after transfection. Decrease of tPA protein expression was monitored by zymography and immunocytofluorescence 72 h after siRNA transfection. Cells were subjected to plasminogen treatment for P36 peptide cleavage 24 h after transfection.

### Quantitative real-time PCR

RNA isolation was performed using the “Qiagen RNeasy™” kit according to the manufacturer's instructions. cDNA was prepared from 100 ng of total RNA by reverse transcription (RT) at 42°C for 45 min using the Maxima First Strand cDNA synthesis kit. DreamTaq Green PCR Master Mix was used for the PCR reaction. The following primers were used: tPA forward primer: 5′-gcaggctgacgtgggagtac-3′ and reverse primer: 5′-ctcctgtgcttggcaaagatg-3′; EEF1A1 forward primer: 5′-ctggagccaagtgctaacatgcc-3′ and reverse primer 5′-ccgggtttgagaacaccagtc-3′. The PCR protocol, was described previously [15]. Melting curve analysis was performed by continuously measuring fluorescence during heating from 55 to 95°C at a transition rate of 0.2°C/s. Product specificity was evaluated by melting-curve analysis and by electrophoresis in 2% agarose gel. Fluorescence was analyzed by the Data Analysis software (Stratagene). Crossing points (Cp or Ct) were established using the second derivative method. The qPCR efficiency was calculated from the slope of the standard curve. Target gene expression levels were normalized to reference gene. The results were calculated using the delta-delta method.

### tPA detection by immunocytofluorescence

24 h after siRNA transfection, SKMEL28 cells were plated on glass slides and incubated in 10% serum-containing medium for 48 h. They were fixed for 5 min with 4% paraformaldehyde. The slides were washed with PBS and saturated in PBS-T with 5% BSA. Cells were then incubated for 1 h at room temperature with an anti-tPA antibody diluted 1/400 in PBS-T with 1% BSA. Slides were washed in PBS and cells were incubated for 30 min with the Alexa-488-conjugated secondary antibody diluted 1/1000 in PBS-T with 1% BSA. For immunocytofluorescence, experiment cells were mounted on slides using Immu-Mount® and studied using a Zeiss LSM 710® confocal laser scanning microscope with the 60x oil-immersion objective (Carl Zeiss microimaging, Germany).

### Molecular dynamics simulations

Molecular dynamic simulations on the different peptides were conducted using the GROMACS simulation package [48]. OPLSAA force field was chosen as the set of parameters for describing the atoms and their interactions [49]. Isolated peptides were placed in boxes with side length varying from 50 Å to 90 Å depending on their size. These values were chosen so that a given peptide would not interact with its image when the periodic boundary conditions are applied. Water (TIP3P model) and counter ions were added prior to the simulations. In order to relax the structures, 500 steps of energy minimization were performed using the steepest descent algorithm. The systems were equilibrated for 500 ps at the temperature of 310 K in the isothermal-isobaric ensemble. The equilibration steps were followed by Molecular Dynamics (MD) simulations carried out for 200 ns, maintaining a pressure of 1 bar (Berendsen algorithm) and a temperature of 310 K (V-rescale algorithm). The Verlet algorithm was used to integrate the equation from classical mechanics in parallel with an integration step of 2 fs since the length of the bonds implicating hydrogen atoms were frozen using the LINCS algorithm. For the computation of the non-bonded interactions, Particle Mesh Ewald (PME) algorithm was used with a cut off at 1.8 Å for the coulombic interactions and a potential-shifting function for van der Waals interactions applied at 1.3 Å and a cut off at 1.4 Å.

The starting structures of the MD simulations, presented in Figure 6A were generated following three different ways:

(i) Each of the peptide was considered in an extented conformation (φ and ψ angles equal to 180° except for the proline residues where (φ, ψ) = (−75°, 150°)). One snapshot was extracted from the F4 MD trajectory in order to create the disulfide bridge between the two cystein residues (in first and sixth position). In order to do that the distance between the S atoms was monitored When it was in agreement with the existence of an S-S bond, we selected the appropriate structure as a new starting conformation. (ii) The I-TASSER server was used to get a set of predicted structures for P19 and F4 peptides [50], (iii) Pepfold server was used to get a second set of predicted structures for the three peptides [51, 52, 53]. Since pepfold allows us to consider the possible creation of a disulfide bridge between the two cystein residues, a second starting conformation presenting an S-S bond was generated in the case of the F4 peptide.

In total, eight starting structures were generated and thus, eight independent MD simulations were performed (three for P19 and F4 peptides and 2 for the S-S bridged F4 peptide).

## SUPPLEMENTARY FIGURE AND TABLES



## References

[1] Yurchenco PD (2011). Basement membranes: cell scaffoldings and signaling platforms. Cold Spring Harb Perspect Biol.

[2] LeBleu VS, Macdonald B, Kalluri R (2007). Structure and function of basement membranes. Exp Biol Med (Maywood).

[3] Kalluri R (2003). Basement membranes: structure, assembly and role in tumour angiogenesis. Nat Rev Cancer.

[4] Suhr F, Brixius K, Bloch W (2009). Angiogenic and vascular modulation by extracellular matrix cleavage products. Curr Pharm Des.

[5] Maquart FX, Siméon A, Pasco S, Monboisse JC (1999). Regulation of cell activity by the extracellular matrix: the concept of matrikines. J Soc Biol.

[6] Monboisse JC, Oudart JB, Ramont L, Brassart-Pasco S, Maquart FX (2014). Matrikines from basement membrane collagens: a new anti-cancer strategy. Biochim Biophys Acta.

[7] Hamano Y, Zeisberg M, Sugimoto H, Lively JC, Maeshima Y, Yang C, Hynes RO, Werb Z, Sudhakar A, Kalluri R (2003). Physiological levels of tumstatin, a fragment of collagen IV alpha3 chain, are generated by MMP-9 proteolysis and suppress angiogenesis via alphaV beta3 integrin. Cancer Cell.

[8] Ricard-Blum S (2011). The collagen family. Cold Spring Harb Perspect Biol.

[9] Salant DJ (2010). Goodpasture's disease—new secrets revealed. N Engl J Med.

[10] Dhar DK, Ono T, Yamanoi A, Soda Y, Yamaguchi E, Rahman MA, Kohno H, Nagasue N (2002). Serum endostatin predicts tumor vascularity in hepatocellular carcinoma. Cancer.

[11] Luo YQ, Li-Juan Yao, Zhao L, Sun AY, Dong H, Du JP, Wu SZ, Hu W (2010). Development of an ELISA for quantification of tumstatin in serum samples and tissue extracts of patients with lung carcinoma. Clin Chim Acta.

[12] Bono P, Teerenhovi L, Joensuu H (2003). Elevated serum endostatin is associated with poor outcome in patients with non-Hodgkin lymphoma. Cancer.

[13] Kiliś-Pstrusińska K, Wikiera-Magott I, Zwolińska D, Kopeć W, Rzeszutko M (2002). Analysis of collagen IV and fibronectin in blood and urine in evaluation of nephrotic fibrosis in children with chronic glomerulonephritis. Med Sci Monit.

[14] Szarvas T, László V, Vom Dorp F, Reis H, Szendröi A, Romics I, Tilki D, Rübben H, Ergün S (2012). Serum endostatin levels correlate with enhanced extracellular matrix degradation and poor patients' prognosis in bladder cancer. Int J Cancer.

[15] Oudart JB, Brassart-Pasco S, Luczka E, Dupont-Deshorgue A, Bellon G, Boudko SP, Bächinger HP, Monboisse JC, Maquart FX, Ramont L (2013). Analytical methods for measuring collagen XIX in human cell cultures, tissue extracts, and biological fluids. Anal Biochem.

[16] Yoshioka H, Zhang H, Ramirez F, Mattei M-G, Moradi-Ameli M, van der Rest M, Gordon MK (1992). Synteny between the loci for a novel FACIT-like collagen locus (D6S228E) and α1(IX) collagen (COL9A1) on 6q12–q14 in humans. Genomics.

[17] Myers JC, Sun MJ, D'Ippolito JA, Jabs EW, Neilson EG, Dion AS (1993). Human cDNA clones transcribed from an unusually high-molecular-weight RNA encode a new collagen chain. Gene.

[18] Myers JC, Yang H, D'Ippolito JA, Presente A, Miller MK, Dion AS (1994). The triple-helical region of human type XIX collagen consists of multiple collagenous subdomains and exhibits limited sequence homology to alpha 1(XVI). J Biol Chem.

[19] Sumiyoshi H, Inoguchi K, Khaleduzzaman M, Ninomiya Y, Yoshioka H (1997). Ubiquitous expression of the alpha1(XIX) collagen gene (Col19a1) during mouse embryogenesis becomes restricted to a few tissues in the adult organism. J Biol Chem.

[20] Inoguchi K, Yoshioka H, Khaleduzzaman M, Ninomiya Y (1995). The mRNA for &alpha;1(XIX) Collagen Chain, a New Member of FACITs, Contains a Long Unusual 3′ Untranslated Region and Displays Many Unique Splicing Variants. The Journal of Biochemistry.

[21] Gerecke DR, Olson PF, Koch M, Knoll JH, Taylor R, Hudson DL, Champliaud MF, Olsen BR, Burgeson RE (1997). Complete primary structure of two splice variants of collagen XII, and assignment of alpha 1(XII) collagen (COL12A1), alpha 1(IX) collagen (COL9A1), and alpha 1(XIX) collagen (COL19A1) to human chromosome 6q12–q13. Genomics.

[22] Khaleduzzaman M, Sumiyoshi H, Ueki Y, Inoguchi K, Ninomiya Y, Yoshioka H (1997). Structure of the human type XIX collagen (COL19A1) gene, which suggests it has arisen from an ancestor gene of the FACIT family. Genomics.

[23] Myers JC, Li D, Bageris A, Abraham V, Dion AS, Amenta PS (1997). Biochemical and immunohistochemical characterization of human type XIX defines a novel class of basement membrane zone collagens. Am J Pathol.

[24] Amenta P.S, Hadad S, Lee MT, Barnard N, Li D, Myers J.C (2003). Loss of types XV and XIX collagen precedes basement membrane invasion in ductal carcinoma of the female breast. J. Pathol.

[25] Toubal A, Ramont L, Terryn C, Brassart-Pasco S, Patigny D, Sapi J, Monboisse JC, Maquart FX (2010). The NC1 domain of type XIX collagen inhibits melanoma cell migration. Eur J Dermatol.

[26] Ramont L, Brassart-Pasco S, Thevenard J, Deshorgue A, Venteo L, Laronze JY, Pluot M, Monboisse JC, Maquart FX (2007). The NC1 domain of type XIX collagen inhibits *in vivo* melanoma growth. Mol Cancer Ther.

[27] Wilman B (1977). Primary structure of the B-chain of human plasmin. Eur J Biochem.

[28] Madison EL, Coombs GS, Corey DR (1995). Substrate specificity of tissue type plasminogen activator. Characterization of the fibrin independent specificity of t-PA for plasminogen. J Biol Chem.

[29] Boudko SP, Zientek KD, Vance J, Hacker JL, Engel J, Bächinger HP (2010). The NC2 domain of collagen IX provides chain selection and heterotrimerization. J Biol Chem.

[30] Kabsch W, Sander C (1983). Dictionary of protein secondary structure: pattern recognition of hydrogen-bonded and geometrical features. Biopolymers.

[31] Liotta LA, Steeg PS, Stetler-Stevenson WG (1991). Cancer metastasis and angiogenesis: an imbalance of positive and negative regulation. Cell.

[32] Monboisse JC, Oudart JB, Brezillon S, Brassart B, Ramont L, Maquart FX, Brassart-Pasco S (2013). Control of tumor progression by extracellular matrix molecule fragments, the matrikines. J Carcinogenes Mutagenes.

[33] Khatib AM, Nip J, Fallavollita L, Lehmann M, Jensen G, Brodt P (2001). Regulation of urokinase plasminogen activator/plasmin-mediated invasion of melanoma cells by the integrin vitronectin receptor alphavbeta3. Int J Cancer.

[34] Meissauer A, Kramer MD, Schirrmacher V, Brunner G (1992). Generation of cell surface-bound plasmin by cell-associated urokinase-type or secreted tissue-type plasminogen activator: a key event in melanoma cell invasiveness *in vitro*. Exp Cell Res.

[35] Fukuda H, Mochizuki S, Abe H, Okano HJ, Hara-Miyauchi C, Okano H, Yamaguchi N, Nakayama M, D'Armiento J, Okada Y (2011). Host-derived MMP-13 exhibits a protective role in lung metastasis of melanoma cells by local endostatin production. Br J Cancer.

[36] Heljasvaara R, Nyberg P, Luostarinen J, Parikka M, Heikkilä P, Rehn M, Sorsa T, Salo T, Pihlajaniemi T (2005). Generation of biologically active endostatin fragments from human collagen XVIII by distinct matrix metalloproteases. Exp Cell Res.

[37] Ma DH-K, Yao J-Y, Kuo M-T, See L-C, Lin K-Y, Chen S-C, Chen JK, Chao AS, Wang SF, Lin KK (2007). Generation of endostatin by matrix metalloproteinase and cathepsin from human limbocorneal epithelial cells cultivated on amniotic membrane. Invest Ophthalmol Vis Sci.

[38] Felbor U, Dreier L, Bryant RA, Ploegh HL, Olsen BR, Mothes W (2000). Secreted cathepsin L generates endostatin from collagen XVIII. EMBO J.

[39] Veillard F, Saidi A, Burden RE, Scott CJ, Gillet L, Lecaille F, Lalmanach G (2011). Cysteine cathepsins S and L modulate anti-angiogenic activities of human endostatin. J Biol Chem.

[40] O'Reilly MS, Boehm T, Shing Y, Fukai N, Vasios G, Lane WS, Flynn E, Birkhead JR, Olsen BR, Folkman J (1997). Endostatin: an endogenous inhibitor of angiogenesis and tumor growth. Cell.

[41] Chen H, Xue L-X, Cao H-L, Chen S-W, Guo Y, Gao WW, Ju SM, Tian HL (2013). Endostatin/collagen XVIII is increased in cerebrospinal fluid after severe traumatic brain injury. Biomed Res Int.

[42] Andreasen PA, Egelund R, Petersen HH (2000). The plasminogen activation system in tumor growth, invasion, and metastasis. Cell Mol Life Sci.

[43] De Vries TJ, van Muijen GN, Ruiter DJ (1996). The plasminogen activation system in melanoma cell lines and in melanocytic lesions. Melanoma Res.

[44] Lauer-Fields JL, Sritharan T, Stack MS, Nagase H, Fields GB (2003). Selective hydrolysis of triple-helical substrates by matrix metalloproteinase-2 and -9. J Biol Chem.

[45] Lauer-Fields JL, Tuzinski KA, Shimokawa Ki, Nagase H, Fields GB (2000). Hydrolysis of triple-helical collagen peptide models by matrix metalloproteinases. J Biol Chem.

[46] Thevenard J, Floquet N, Ramont L, Prost E, Nuzillard J-M, Dauchez M, Yezid H, Alix AJ, Maquart FX, Monboisse JC, Brassart-Pasco S (2006). Structural and antitumor properties of the YSNSG cyclopeptide derived from tumstatin. Chem Biol.

[47] Thevenard J, Ramont L, Devy J, Brassart B, Dupont-Deshorgue A, Floquet N, Schneider L, Ouchani F, Terryn C, Maquart FX, Monboisse JC, Brassart-Pasco S (2010). The YSNSG cyclopeptide derived from tumstatin inhibits tumor angiogenesis by down-regulating endothelial cell migration. Int J Cancer.

[48] Van Der Spoel D, Lindahl E, Hess B, Groenhof G, Mark AE, Berendsen HJC (2005). GROMACS: fast, flexible, and free. J Comput Chem.

[49] Jorgensen WL, Tiradorives J (1998). The Opls Potential Functions for Proteins - Energy Minimizations for crystals of cyclic peptides and crambin. J. Am. Chem. Soc.

[50] Zhang Y (2008). I-TASSER server for protein 3D structure prediction. BMC Bioinformatics.

[51] Maupetit J, Derreumaux P, Tufféry P (2010). A fast method for large-scale de novo peptide and miniprotein structure prediction. J Comput Chem.

[52] Maupetit J, Derreumaux P, Tufféry P (2009). PEP-FOLD: an online resource for de novo peptide structure prediction. Nucleic Acids Res.

[53] Thévenet P, Shen Y, Maupetit J, Guyon F, Derreumaux P, Tufféry P (2012). PEP-FOLD: an updated de novo structure prediction server for both linear and disulfide bonded cyclic peptides. Nucleic Acids Res.

