# Supplementary material for: Plasmin releases the anti-tumor peptide from the NC1 domain of collagen XIX
Source: Oncotarget. 2015 Jan 21;6(6):3656–68. doi: 10.18632/oncotarget.2849 (PMC4414144; doi:10.18632/oncotarget.2849)
Supplement: Supplementary file 1 [file oncotarget-06-3656-s001.pdf]

SUPPLEMENTARY FIGURE AND TABLES

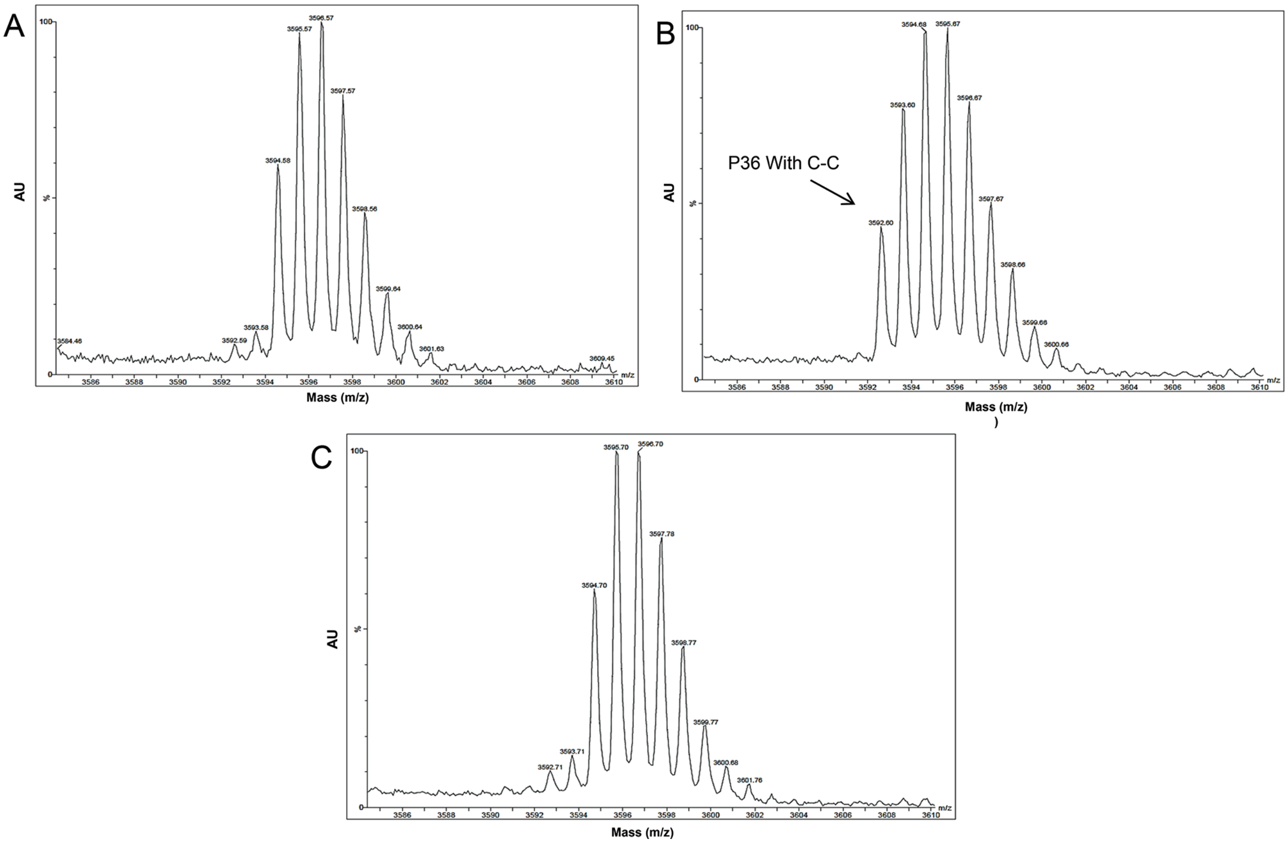

Supplementary Figure S1: NC1(XIX) domain of collagen XIX makes disulfide bond in solution. **(A)** MALDI-ToF MS analysis showing that P36 peptide in solution at T0 presented no disulfide bond. **(B)** MALDI-ToF MS analysis at 1 h demonstrating that the P36 peptide of collagen XIX made a disulfide bond in its NC1(XIX) domain over the time. **(C)** MALDI-ToF MS analysis showing that in the presence of dithiothreitol (DTT), the disulfide bond disappeared

Supplementary Table S1: β-turn analysis of the trajectories

Peptides	Starting structure	Sequence of typeI turn above threshold
P19	**Elongated**	SHAH ; HAHQ ; QRTG
	**Itasser prediction**	NPED ; LYPV ; VSHA ; QRTG
	**Pepfold prediction**	NPED ; PVSH ; VSHA ; AHQR ; RTGG
F4 without disulfide bridge	**Elongated**	SHAH
	**Itasser prediction**	NPED ; VSHA ; SHAH ; HAHQ
	**Pepfold prediction**	NPED ; SHAH
F4 with disulfide bridge	**From Elongated start**	NPED ; PEDC
	**Pepfold prediction**	NPED ; DCLY ; PVSH ; VSHA ; SHAH ; HAHQ ; AHQR

Supplementary Table S2: Sequences of the peptides used in the study

Names	Peptide sequences	Substitutions
P19	^{1124}NPEDCLYPVSHAHQRTGGN^{1142}	
P36	^{1107}SPGAPGPQGPPGPSGRCNPEDCLYPVSHAHQRTGGN^{1142}	
P36G^{1122}	^{1107}SPGAPGPQGPPGPSG**G**CNPEDCLYPVSHAHQRTGGN^{1142}	Arg^{1122} → Gly
P36W^{1121}	^{1107}SPGAPGPQGPPGPS**W**RCNPEDCLYPVSHAHQRTGGN^{1142}	Gly^{1121} → Trp
P36W^{1120}	^{1107}SPGAPGPQGPPGP**W**GRCNPEDCLYPVSHAHQRTGGN^{1142}	Ser^{1120} → Trp
P36W^{1123}	^{1107}SPGAPGPQGPPGPSGR**W**NPEDCLYPVSHAHQRTGGN^{1142}	Cys^{1123} → Trp
P36W^{1124}	^{1107}SPGAPGPQGPPGPSGRC**W**PEDCLYPVSHAHQRTGGN^{1142}	Asp^{1124} → Trp
